# Belladonna in Labor

**Published:** 1891-08

**Authors:** 


BELLADONNA IN LABOR.—Dr. Aasher, of Lithgow, New South Wales, advises the use of belladonna in the early stages of labor, having found it of immeasurable benefit, saving considerabte pain to the patient and materially diminishing the expected period of the labor. In primiparae, after a prolonged period of pains of more or less intensity, and with but little dilatation of the os, as well as in the more intense condition of a completely rigid os, where, with extreme contractions, no dilatation whatever occnrs, he has given large doses of belladonna with marked effect. He usually prescribes a reliable tincture of belladonna in doses of twenty to thirty minims every hour, or oftener; and satisfactory dilatation usually follows the first or second draught.—*Australasian Med. Gazette.*

